# Monitoring Nitrogen Leaching for the Evaluation of the Dutch Minerals Policy for Agriculture in Clay Regions

**DOI:** 10.1100/tsw.2001.310

**Published:** 2001-11-03

**Authors:** Dico Fraters, Leo J.M. Boumans, Ton C. van Leeuwen, Wim D. de Hoop

**Affiliations:** National Institute of Public Health and the Environment, Bilthoven, The Netherlands

**Keywords:** groundwater quality, clay regions, nitrate, ammonium, total nitrogen, Kjeldahl-nitrogen, tile drains, farm practices, nitrogen surpluses, nitrate leaching

## Abstract

This paper presents the results of the Dutch monitoring program for agriculture in the clay regions for the period 1996–2000 and evaluates the monitoring strategy. A wide range of farms (25 to 85%) had a NO_3_-N concentration in tile drainwater higher than the EU standard of 11.3 mg/l. The low figure is related to wet winters; the high, to dry winters. Arable farms are more prone to NO_3_ leaching than dairy farms. On arable farms, about 25% of the N surplus leached to groundwater and tile drainwater, on dairy farms this was about 15%. N in tile drainwater has shown to be the best indicator for monitoring the effects of farming practice changes in the clay regions. The average NO_3_-N concentration in tile drainwater was 18.8 and 3.2 mg/l in borehole water on farms where both were monitored. It is known that N use has a relationship with NO_3_ in tile drainwater and not with NH_4_ and organic N. The presented results indicate that crop rotation and precipitation strongly influence NO_3_ concentration in tile drainwater.

### INTRODUCTION

In many European countries, $NO_3^-$ leaching from agricultural land has caused great concern for more than 20 years. Numerous examples of increasing $NO_3^-$ concentrations in groundwater were reported in the early 1980s[1]. The excessive use of nutrients in agriculture has been concluded by the European Union (EU) to constitute an environmental risk. In 1991, all member states adopted the Nitrate Directive[2], which sets goals both for preventing pollution and decreasing existing $NO_3^-$ concentrations in groundwater and surface water. Member states are obliged to establish action programs and to monitor its efficacy.

In 1987, the National Monitoring Program for Effectiveness of the Minerals Policy (LMM), aimed at monitoring the effects of the Dutch minerals policy, was initiated in the sandy regions in the Netherlands. Three soil type regions are distinguished within the LMM: sand, clay, and peat[3,4]. In 1993, the LMM was extended to the clay regions and to the peat regions in 1995. Within the LMM, both farm practices and upper groundwater quality are monitored at farm level. Upper groundwater is defined as the most recently formed groundwater (saturated zone, including tile drainwater) or soilwater (vadose zone) occurring within 5 m of the soil surface, but unrecoverable by crops either directly or by capillary rise.

In the 1993–1996 period a scouting program was carried out on 18 farms in the clay regions[5]. The total yearly average $NO_3^-$-N concentration in tile drainwater for all farms ranged in this period from 4.9 to 11.1 mg/l. The percentage of farms having a yearly average $NO_3^-$ concentration of higher than the EU standard of 11.3 mg/l ranged between 15 and 40%.

Here, we will discuss the evaluation of the follow-up scanning program on 99 farms in the 1996–2000 period (Fig. 1) to

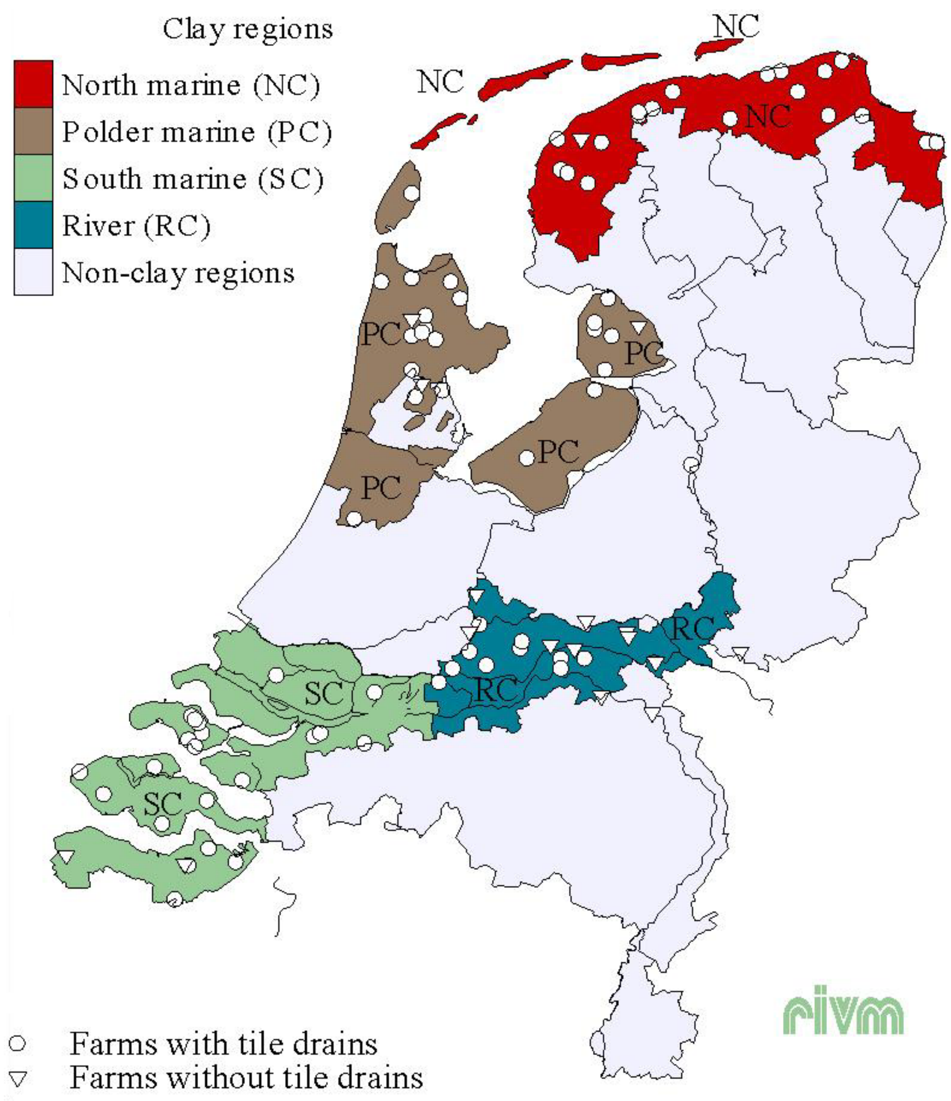

**FIGURE 1**. Participating farms in the 1996–2001 scanning program in the clay regions in the Netherlands.

define the monitoring strategy for the next program starting in 2002. The presented research was carried out in two different subprograms: a tile drainwater sampling subprogram and a borehole water sampling subprogram.

### EXPERIMENTAL METHODS

#### The Clay Regions

In the Netherlands thirteen regions are distinguished on the basis of soil type and agronomic characteristics. Four regions have mainly clay soils: these are the North marine clay region (NC), the Lake IJssel Polders and North Holland (PC), the Southwest marine clay region (SC), and the River clay region (RC), see Fig. 1. The agricultural area in the clay regions amounts to 763,000 ha, which is about 38% of the total agricultural area in the Netherlands. Grassland comes to 281,000 ha, while maize occupies 42,000 ha, and other crops 440,000 ha. There are about 33,000 farms in this area.

The NC, SC, and PC regions have marine clays. Sedimentation occurred in tidal marshes[6]. Surface level ranges from around sea level (1 m below to 1.5 m above; NC and SC) to 3 to 6 m below (PC)[6]. Soils are Gleyo-Calcaric, Gleyo-Eutric Fluvisols, and Mollic Gelysols[7]. The RC is characterized by Holocene and late Pleistocene Rhine and Meuse River deposits. Surface level ranges from an average of 13 m above sea level in the eastern part to 1.5 m above sea level in the western part. Soil types are Gleyo-Eutric Fluvisols and Fluvi-Calcic Cambisols.

#### Hydrological Characteristics[5,7]

The Netherlands has a cool, marine, temperate climate. The soil moisture regime is udic and the soil temperature regime mesic. The average precipitation per year in the clay region for the 1970–2000 period was 800 mm (see Fig. 2, upper graph). In the summer months, the potential evapotransperation exceeds the precipitation (see Fig. 2, lower graph), and the groundwater table is usually found below tile drain level. In winter, the storage of water is replenished and on average 280 mm of the precipitation percolates into the soil.

Tile drainage system installation started some 100 years ago. Tiles are usually installed at a depth of roughly 1 m below surface level, under a slope of 1:1000, and spaced 10 m apart. About 50% of the precipitation surplus is estimated to be discharged by tile drains, while the rest is mainly groundwater recharge. Usually, only the reclaimed soils in the PC discharge 80 to 100% by tiles.

### Monitoring Programs

The upper meter of groundwater on 60 farms was sampled once via boreholes in the 1996–1997 period (borehole subprogram). Tile drainwater was sampled on 69 farms for 1 to 4 years between 1996 and 2000 (tile drain subprogram). Of the 69 farms, 30 also participated in the borehole subprogram, so that 99 different farms participated in this scanning program.

Farms were randomly selected after stratification of farms participating in the Farm Accountancy Data Network (FADN) of the Agricultural Economics Research Institute (LEI). The FADN produces a representative test sample of 1500 farms where farm management and economic aspects are monitored for 5 to 6 years[8]. The number of farms selected for the borehole water and tile drain subprograms is given in Table 1. Additional selection criteria comprise a minimum farm acreage of 10 ha, and for the tile drain subprogram at least 25% of the area is drained by tile drains. Of the 60 farms in the borehole subprogram, about 15% of the farms in the marine clay regions, and 55% in the river clay region showed less than 25% of the acreage drained by tile drains.

### Sampling and Data Collection

On each farm, 16 tile drains and or locations for sampling via boreholes were selected using a standard procedure[3]. Tiles were sampled four times during the drainage season (October–April). Samples were stored in a cooler and transported to the laboratory within 24 h of sampling. Upper groundwater via boreholes was sampled on arable farms in March–April 1996, on dairy farms situated on river clay in July–September 1996, and on dairy farms on marine clay in May-June 1997. A borehole was made with an auger up to 1 m below the groundwater table, followed by the placement of a sampling probe fitted with a PVC screen. Shingle was poured into the hole to a height of 0.8 m from the bottom,

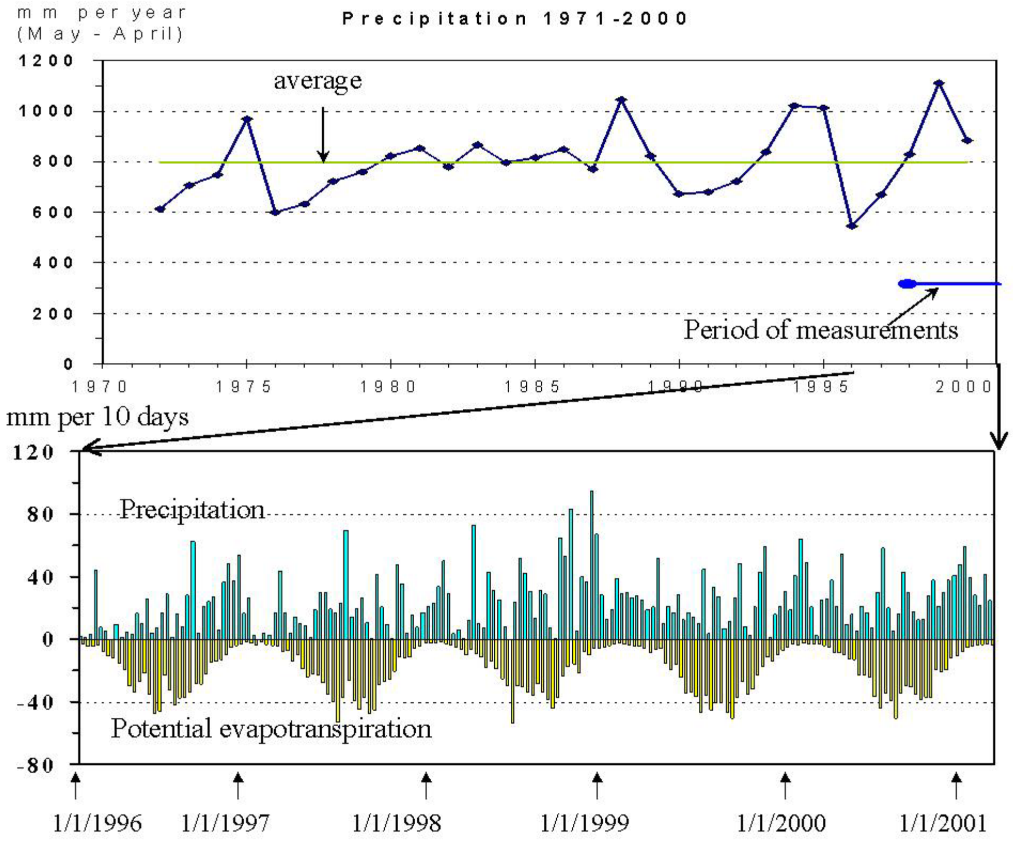

**FIGURE 2**. Precipitation per year (upper graph) and precipitation and potential evapotranspiration per 10 days (lower graph). Data from the Royal Netherlands Meteorological Institute.

**TABLE 1**
**Number of Farms in Tile Drain and Borehole Water (Between Brackets) Subprogram per Combination of Farm Type and Clay Region (30 Farms Participated in both Programs)**

| | Clay Areas | | | | |
|---|---|---|---|---|---|
| **Farm type** | **North** | **Polders** | **South** | **River** | **Total** |
| Arable | 7 (5) | 11 (7) | 14 (8) | 0 (0) | 32 (20) |
| Dairy | 13 (12) | 7 (5) | 7 (3) | 6 (20) | 33 (40) |
| Mixed | 3 (0) | 1 (0) | 0 (0) | 0 (0) | 4 (0) |
| Total | 23 (17) | 19 (12) | 21 (11) | 6 (20) | 69 (60) |

and the hole sealed with 0.3 m of Bentonite just on top of the shingle to impede water movement down the disturbed hole. Next, the hole was filled with excavate soil in an in-depth sequence. At the end of the day, after placement of all probes, boreholes were pre-emptied using a suction pump. After a few days, samples were taken after rinsing the sampling tubes by pumping up 0.5 l groundwater. Groundwater was then directly filtered over a 0.45-µm filter (cellulose-nitrate), acidified to pH < 2 (18 M $H_2SO_4$ proanalyze for N compounds), and stored at 4°C prior to analysis.

Data on soil type and groundwater regime class distribution per farm were derived from the map described in an earlier publication[3]. Drainage classes are based on groundwater regimes classes (Gt)[9]. Imperfectly drained soils are soils with Gt I, II, and III; moderately drained soils are soils with Gt IV, V, and VI; well-drained soils with Gt VII and VIII.

### Chemical Analyses

Electric conductivity (EC), pH, and $NO_3^-$ (Nitrachek Reflectometer) were determined in individual samples in the laboratory (tile drain) or directly in the field (boreholes). The tile drain samples were mixed up to one to four samples per farm per round; mixed samples were filtered over a 0.45-µm filter and directly analyzed or acidified to pH < 2 and stored at 4°C up to analysis. Borehole water samples mixed up to four samples per farm were analyzed. Mixed drain and borehole water samples were analyzed. $NO_3^-$ was analyzed by ion chromatography using Dionex system 2000i. Kjeldahl-N and $NH_4^+$ were determined photometrically with Flow Injection Analyses (FIA) (Aquatec Analyzer, Tector type 5400), and Kjeldahl-N was determined after destruction with sulfuric acid using Foss Tecator tablets with a catalyst. Organic N concentration was calculated by subtracting $NH_4^+$-N concentration from Kjeldahl-N concentration.

### Statistical Methods

Most of the statistical analyses were performed with annual farm average values based on laboratory result using MINITAB[10]. Differences in concentrations between clay regions, farm type, and year were analyzed using Tukey's pairwise comparison with a family error rate of 0.05. Differences between borehole and tile drainwater in the 1996–1997 period were analyzed using a T-test for mean and a sign test for median of pairwise differences, where differences were assumed to be significant at $p < 0.05$.

The variability in $NO_3^-$ concentrations is attributed to the different farms, different sampling years and interaction with farms, different drains within a farm and interaction with year, different sampling rounds within a specific farm and year, and different drains within a specific farm and year. The estimates of these variance components of $NO_3^-$ in tile drain water was performed with individual tile drain data (Nitracheck) using the Residual Maximum Likelihood method (REML) of GENSTAT[11].

The influence of water flow rate at sampling on the $NO_3^-$ concentration in tile drainwater was performed with regression analyses of difference between flow-weighted mean $NO_3^-$ concentrations and nonweighted averages using GENSTAT [11].

## RESULTS AND DISCUSSION

### Farm Practices

Based on FADN, Table 2 shows the mineral accounts of major inputs and outputs for the three categories of farms in the clay regions for both the borehole subprogram (1996–1997) and tile drain subprogram (1996–2000.).

The inflow of N was due to the acquisition of farm products such as fertilizer, animal manure, and fodder; and by atmospheric deposition, mineralization, and binding of N (from such leguminosae as clover). In this context, manure that is produced and applied on the farm is part of internal flux and consequently does not belong to inflow on the farm scale. The outflow of N deals with agricultural products, e.g., milk, livestock, roughage, and manure.

Fertilizer and manure are generally the most important inputs for arable farms. In addition to fertilizer fodder (roughage and concentrates) is also an important input on dairy farms. Average N surplus appears to be higher for dairy farms than for arable farms. Farms on which borehole water has been sampled attained higher surpluses than farms participating in the drainwater program. We must emphasize that this approach bases the average mineral accounts on a different number of farms for different years. In practice, N surpluses on farm level vary in time. Fig. 3 shows the change in average N surplus on arable and dairy farms

**TABLE 2**
**Average N Account (kg/ha of Farmland, with Respect to the Year Prior to Sampling) for Arable, Dairy, and Mixed Farms on Marine and River Clay in the Netherlands for both the Borehole Water Subprogram (1996–1997) and Tile Drainwater Subprogram (1996–2000)[1]**

| | Borehole Subprogram | | | Tile Drain Subprogram | | | |
|---|---|---|---|---|---|---|---|
| | Arable Marine | Dairy Marine | Dairy River | Arable Marine | Dairy Marine | Mixed Marine | Dairy River |
| Artificial fertilizer | 185 (38) | 323 (84) | 245 (64) | 170 (47) | 281 (102) | 210 (54) | 221 (49) |
| Manure | 88 (65) | 1 (5) | 41 (42) | 103 (82) | 5 (13) | 0 (0) | 7 (17) |
| Fodder | 7 (22) | 109 (50) | 134 (70) | 0 (7) | 110 (58) | 17 (41) | 127 (71) |
| Other[2] | 48 (6) | 53 (15) | 84 (58) | 52 (24) | 61 (30) | 111 (140) | 141 (70) |
| Input | 328 (82) | 486 (112) | 504 (113) | 327 (100) | 461 (130) | 338 (128) | 498 (115) |
| Agricultural products | 127 (21) | 86 (19) | 85 (21) | 126 (31) | 93 (29) | 130 (93) | 115 (24) |
| Manure | 0 (0) | 9 (12) | 9 (24) | 0 (0) | 14 (25) | 0 (0) | 14 (25) |
| Output | 127 (7) | 94 (21) | 93 (45) | 126 (31) | 107 (46) | 130 (93) | 129 (39) |
| Surplus | 201 (84) | 392 (102) | 411 (80) | 201 (96) | 350 (104) | 208 (96) | 364 (99) |

[1] Values in parentheses show standard deviation.
[2] Mainly atmospheric deposition, further some mineralization and binding of nitrogen.

in the clay regions during the 1989–2000 period (for all available farms participating in the FADN on the basis of representation).

### N in Groundwater

The median total N concentration in borehole water was 7.9 mg/l; this consisted of 47% $NO_3^-$, 29% $NH_4^+$, and 24% organic N. In tile drainwater, the median total N concentration was 12.1 mg/l. $NO_3^-$ also forms the main N compound (77%), with 8% consisting of $NH_4^+$, and 15% organic N. Similar concentration levels and ratios for N compounds are found in tile drainwater from swards in Ireland[12]. Of the farms, 10% had a higher $NO_3^-$-N concentration in borehole water than the EU standard of 11.3 mg/l. In the sandy regions, 95% of farms averaged higher during 1992 to 1995[3] than the EU standard. About 46% of the farms had a higher $NO_3^-$-N concentration in tile drainwater than the EU standard, but this varied annually from 25 to 85% (see Fig. 4). On average, this is somewhat higher than in the 1992–1996 period.

### Relationship of N in Groundwater and Farm Practices

No significant differences in concentrations of N components were found between farm type (arable vs. dairy farming) or clay type (marine vs. river clay); see Table 3. Only the organic N concentration in tile drainwater on arable farms was slightly, but significantly, lower than on dairy farms.

The average N leaching to groundwater and surface water can be roughly estimated, using the mean precipitation surplus of 344 mm/year and mean total N concentration in tile drain water for the 1996–2000 period. The average amount of N leaching was 51 kg/ha for arable farms, 43 kg/ha for dairy farms and mixed farms on marine clay, and 59 kg/ha for dairy farms on river clay.

Using the N surpluses from Table 2, the apparent leaching of the N surplus is 25% for arable farms on marine clay, 13% for dairy and mixed farms on marine clay, and 16% for dairy farms on river clay. This higher leaching percentage for arable land is due to arable land being more prone to leaching than grassland. The cause of this phenomenon is dual. First, arable crops cause higher leaching than grass[13]. Second, arable farms had better-drained, sandier clay soils, while dairy farms had less well-drained and often peatier clay soils, see Table 4. The former soils are more prone to leaching than the latter[9,14].

### Relationship between N in Tile Drainwater and Sampling

Differences in year-mean $NO_3^-$ concentration between tile drains on a farm are larger than differences of the period-mean $NO_3^-$ concentration (average of all years) between tile drains on a farm (see Table 5). This difference between short (1 year) and long-term (4 years) means $NO_3^-$ concentrations of farm drains could be due to crop rotation and/or differences in groundwater travel times. For example, it is known that growing potatoes causes a higher leaching than sugar beets[13]. If it is assumed that except for crops all other factors are the same, within a year there will be differences in $NO_3^-$ concentrations between drains. However, in the end, there will be no differences between drains in the average $NO_3^-$ concentration over all years, because crops are rotated. The hypothesis of crop rotation is supported by the fact that arable farms have the largest differences between short-term and long-term variance. Differences in water travel time from

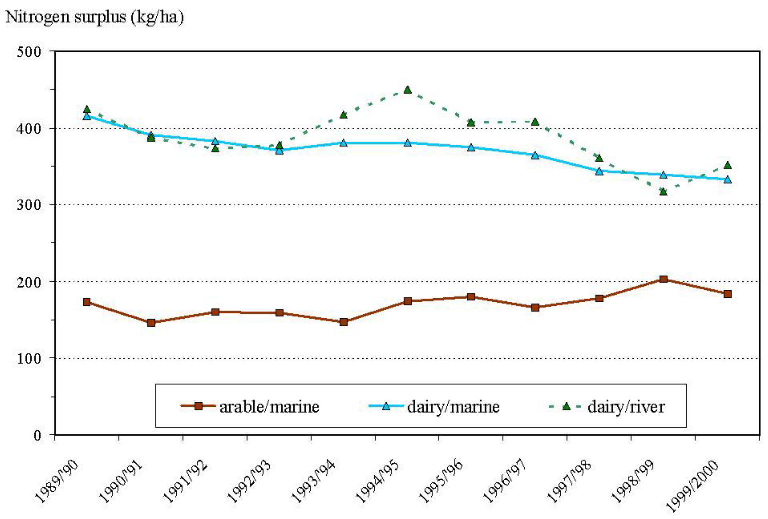

**FIGURE 3**. Average N surplus of FADN farms in the clay regions in the 1989–2000 period.

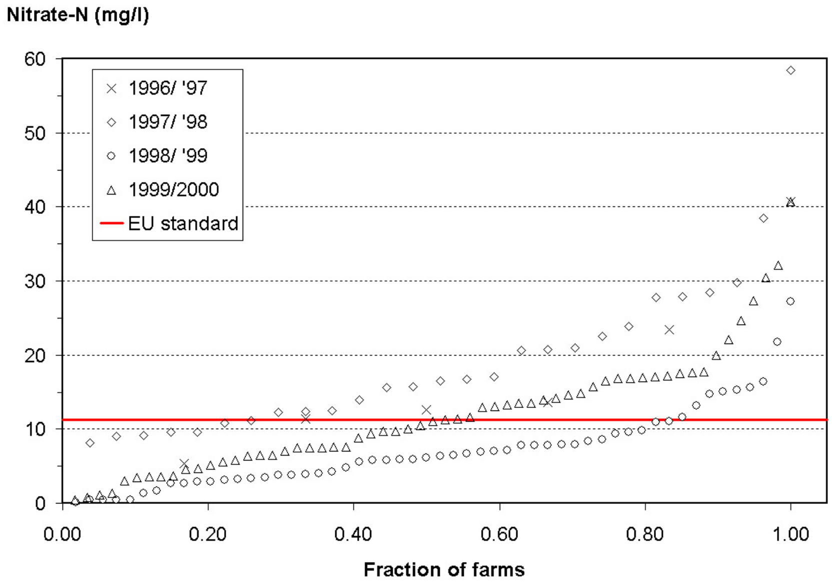

**FIGURE 4**. $NO_3^-$-N in drainwater in 1996–1999; average nitrate concentrations on farms ranked per year.

### TABLE 3
## N Concentration (mg/l) in Borehole Water (1996–1997) and Tile Drainwater (1996–2000) on Arable, Dairy, and Mixed Farms in Marine and River Clay Regions in the Netherlands[1]

| | Borehole Subprogram | | | Tile Drain Subprogram | | | |
|---|---|---|---|---|---|---|---|
| | Arable Marine | Dairy Marine | Dairy River | Arable Marine | Dairy Marine | Mixed Marine | Dairy River |
| Number of farms | 20 | 20 | 20 | 32 | 27 | 4 | 6 |
| Nitrate-N | 4.1[a] | 4.5[a] | 5.4[a] | 12.7[b] | 9.9[b] | 6.9[ab] | 14.5[b] |
| Ammonium-N | 2.4[a] | 3.3[a] | 1.3[a] | 0.59[a] | 1.1[a] | 1.1[a] | 0.44[a] |
| Organic-N | — | 1.1[a] | — | 1.4[a] | 1.9[b] | 1.9[ab] | 2.2[b] |

[1] Non-matching letter within row show significant differences in concentration.

**TABLE 4**
**Farm Average Soil and Drainage Characteristic (Fraction of Acreage) of Arable, Dairy, and Mixed Farms in Marine and River Clay Regions in the Netherlands**

| | Borehole Subprogram | | | Tile Drain Subprogram | | | |
|---|---|---|---|---|---|---|---|
| | Arable Marine | Dairy Marine | Dairy River | Arable Marine | Dairy Marine | Mixed Marine | Dairy River |
| *Soil type* | | | | | | | |
| sand | 0.05 | 0.07 | 0.01 | 0.05 | 0.06 | 0.22 | — |
| clay | 0.95 | 0.92 | 0.99 | 0.95 | 0.83 | 0.75 | 1.00 |
| peat | — | 0.01 | — | — | 0.11 | 0.03 | — |
| *Drainage class* | | | | | | | |
| Imperfect | 0.10 | 0.18 | 0.36 | 0.09 | 0.26 | 0.11 | 0.45 |
| Moderate | 0.76 | 0.75 | 0.59 | 0.84 | 0.70 | 0.89 | 0.52 |
| Good | 0.14 | 0.07 | 0.05 | 0.07 | 0.05 | — | 0.03 |

**TABLE 5**
**Estimated Variance Components[1] for $NO_3^-$ in Tile Drainwater on Farms in the Clay Regions of the Netherlands for 1996–2000 for all Farms and per Farm Type**

| | Farm Type | | | |
|---|---|---|---|---|
| Source | All Marine | Arable Marine | Dairy River | Dairy River |
| Between farms | 445[ac] | –72[a] | 447[ac] | 371[abc] |
| Between years | 371[abc] | 234[abd] | 565[ac] | 546[abc] |
| Interaction of combination of farm and years | 579[a] | 491[abd] | 574[a] | 934[abc] |
| Between drains on a farm | 447[a] | 287[b] | 427[a] | 1923[b] |
| Between drains on a farm within a year | 1056[b] | 1648[c] | 1061[b] | 546[c] |
| Between rounds on a farm within a year | 295[c] | 381[b] | 153[c] | 513[c] |
| Residual variance | 502[a] | 661[a] | 461[a] | 541[c] |

[1] Non-matching letter within columns shows significant differences in variance components.

the surface to the drains, in case there are different surpluses between years, will have a similar effect. The consequence for monitoring is the number of drains sampled per farm can be limited in case the farm is monitored for several years compared with a single sampling year.

The differences between round mean $NO_3^-$ concentrations of each separate farm within a year are relatively small. Therefore, increasing the number of rounds pays off less than increasing the number of drains on a farm. Sampling 16 tile drains per farm, 4 times a year instead of, e.g., 4 tile drains,16 times a year, is a judicious choice.

### Relationship Between $NO_3^-$ in Tile Drainwater and Flow Rate at Sampling

At the farm level, no relationship was found between flow rate at sampling of and $NO_3^-$ concentration in tile drainwater within a round (same date of sampling). Thus, flow-weighted mean $NO_3^-$ concentrations per farm, per round are clearly similar to nonweighted averages. Therefore, water samples from different tile drains from one farm within one round can be mixed to save on analysis cost.

Differences between flow-weighted mean $NO_3^-$ concentrations and nonweighted averages of different farms (all rounds) and of different rounds (all farms) are small, but significant: 0.72 and 0.63 mg/l as N, respectively.

### Effects of Precipitation on $NO_3^-$ in Tile Drainwater

$NO_3^-$ concentrations differed significantly from year to year (see Fig. 4). Concentrations in 1997–1998 (October 1997–April 1998) were the highest, and in 1998–1999, the lowest. We propose that this is an effect of the variation in precipitation—higher precipitation resulting in more dilution and lower concentrations (see Fig. 2). A similar effect of precipitation on $NO_3^-$ in upper groundwater has been shown for the sandy regions[3].

$NO_3^-$ losses to drainage water are reported to be greater in an autumn following a dry summer[15,16]. Realizing that we only have data for 4 years, and although there is a (nonsignificant) correlation between precipitation in the summer and the following winter, we found that $NO_3^-$ concentrations were significantly related with precipitation in winter, not in summer. Precipitation was not found to have an effect of on $NH_4^+$ or organic N concentration.

This phenomenon's consequences for monitoring the effectiveness of the action program is that it will take more time to detect an effect. This counteracts the fact that effects of farming practice occur first in recently formed groundwater. The data of this program will be used to develop a method for adjusting measured data for differences in precipitation between years, as has been done for the sandy regions[3].

### An Environmental Indicator for Changes in Farming Practices

Others have shown[12,16] that there is a relationship between N use and $NO_3^-$ in tile drainwater, while there is no relationship between N use and $NH_4^+$ or organic N in tile drainwater. This research made a reasonable case for the strong and quick response of $NO_3^-$ in tile drain to crop rotation (Table 5) and precipitation (Fig. 4).

Surface water quality is of higher importance than groundwater quality in the clay areas in the Netherlands, where salinity largely impedes domestic use of groundwater. Tile drainwater is an important source for surface waters.

Concentrations of N compounds in tile drainwater and borehole water differed significantly on the 30 farms where both borehole and tile drainwater were sampled, which is a confirmation of the general impression shown in Table 3. On average, $NO_3^-$-N concentration in tile drainwater was 15.6 mg/l higher than in borehole water: 18.8 vs. 3.2 mg/l. The lower $NO_3^-$ concentrations in borehole water could be a combination of a longer travel times and higher denitrification losses. Thus, monitoring tile drainwater is more suitable than borehole water for following $NO_3^-$ concentration changes.

In the Netherlands, most of the farms in the clay regions are drained by tiles. This and the above given arguments make $NO_3^-$ in tile drainwater the best indicator for monitoring farming practice effects.

### CONCLUSIONS

Based on results from the first 4 years of the upper groundwater monitoring program in the clay regions in the Netherlands, we conclude $NO_3^-$ concentration in tile drainwater to be a better indicator of farming practice changes in clay regions than $NH_4^+$, organic N in tile drainwater, or than one of the N compounds in the upper meter of groundwater sampled via boreholes. Tile drainwater samples from a sampling round of one farm can be mixed to save on analysis cost. The present strategy of sampling 16 tile drains per farm per round and 4 rounds per year is a judicious choice. Proneness to leaching of $NO_3^-$ is almost twice as high from arable land as from grasslands. $NO_3^-$ concentration in tile drainwater depends on the amount of winter precipitation; this impedes the swift monitoring efficacy of action programs.

### ACKNOWLEDGEMENTS

The authors would like to express their gratitude to Hans Bronswijk, Jaap Willems, and Ruth de Wijs-Christensen for their suggestions and critical comments on drafts of this paper. We also thank L.F.L. Gast, R. Jeths, H.L.J. van Maaren, N. Masselink, H.F. Prins, and others who assisted in the data collection.

| | | |
|---|---|---|
| Received: | July | 9, 2001 |
| Revised: | October | 5, 2001 |
| Accepted: | October | 15, 2001 |
| Published: | November | 3, 2001 |
